# R-Loop Mediated Transcription-Associated Recombination in *trf4*Δ Mutants Reveals New Links between RNA Surveillance and Genome Integrity

**DOI:** 10.1371/journal.pone.0065541

**Published:** 2013-06-07

**Authors:** Sandra Gavaldá, Mercedes Gallardo, Rosa Luna, Andrés Aguilera

**Affiliations:** 1 Departamento de Biología Molecular, Centro Andaluz de Biología Molecular y Medicina Regenerativa CABIMER, Universidad de Sevilla, Seville, Spain; 2 Departamento de Genética, Universidad de Sevilla, Seville, Spain; Chang Gung University, Taiwan

## Abstract

To get further insight into the factors involved in the maintenance of genome integrity we performed a screening of *Saccharomyces cerevisiae* deletion strains inducing hyperrecombination. We have identified *trf4*, a gene encoding a non-canonical polyA-polymerase involved in RNA surveillance, as a factor that prevents recombination between DNA repeats. We show that *trf4*Δ confers a transcription-associated recombination phenotype that is mediated by the nascent mRNA. In addition, *trf4*Δ also leads to an increase in the mutation frequency. Both genetic instability phenotypes can be suppressed by overexpression of RNase H and are exacerbated by overexpression of the human cytidine deaminase AID. These results suggest that in the absence of Trf4 R-loops accumulate co-transcriptionally increasing the recombination and mutation frequencies. Altogether our data indicate that Trf4 is necessary for both mRNA surveillance and maintenance of genome integrity, serving as a link between RNA and DNA metabolism in *S. cerevisiae*.

## Introduction

Maintenance of genome integrity is critical for cell homeostasis. Cells posses multiple mechanisms such as specific DNA repair pathways or cell cycle checkpoints to deal with DNA damage and the resulting genetic instability commonly associated with cancer and several genetic disorders [1]. Genomes are exposed to the action of physical and chemical agents, and metabolic processes that can cause lesions in the DNA. One such process is transcription, which has been established as an inducer of genome instability. Recombination and mutation frequencies are enhanced by transcription, leading to transcription-associated recombination (TAR) and transcription-associated mutation (TAM) [2], [3]. Key to understanding how transcription increases genomic instability is the fact that single-stranded DNA (ssDNA) is chemically more unstable than double-stranded DNA (dsDNA). Transcription itself and changes in topology and chromatin conformation associated with it may increase the probability of the occurrence of ssDNA. Consistently, DNA-damaging agents show a synergistic effect with transcription in the induction of recombination in yeast [4], and mutation rates correlate with the strength of transcription and superhelical stress [5]. In addition to a major ssDNA accessibility, transcription associated genomic instability could also be the result of the collision between the transcription and replication machineries [6], [7]. A possible intermediate of transcription-associated genomic instability is an R-loop structure consisting of a RNA:DNA hybrid that displaces the non-template ssDNA strand. R-loops are transcription by-products rarely formed in the cell but they accumulate in a number of transcription and mRNP mutants with a genetic instability phenotype [8].

During transcription, the nascent pre-mRNA associates with mRNA-binding proteins and undergoes a series of processing steps resulting in an export competent mRNA ribonucleoprotein complexes (mRNP) [9], [10]. Emerging evidence suggest that when mRNP biogenesis does not occur properly the RNA can hybridize with the DNA template, forming R-loops that would hinder transcription elongation and block replication. One of the best studied examples is the THO complex, which functions at the interface transcription-mRNA export. Mutations in THO lead to a transcription-associated hyperrecombination phenotype partially suppressed by overexpression of RNase H, an enzyme that degrades the RNA strand of DNA:RNA hybrids [11]. Moreover, in these mutants genome instability is exacerbated by the action of the human cytidine deaminase AID that acts on the displaced ssDNA of R-loops [12], [13]. Similar R-loop-dependent co-transcriptional genome instability is observed in mammalian and chicken DT40 cells depleted of the ASF/SF2 splicing factor [14]. More recently, mutations in topoisomerase I, SenI/SENATAXIN and Sin3 have also been reported to cause genome instability via a common mechanism [15], [16], [17], [18]. In addition, a number of RNA processing factors have been shown to be relevant for the maintenance of genome integrity by preventing R-loop accumulation by different genetic and cellular approaches in yeast and human cells [17], [19], [20].

In *Saccharomyces cerevisiae,* screenings based on marker stability provide a powerful approach for studying genes that preserve genome structure [21], [22]. These screenings exploit the use of artificial chromosome (YAC) and endogenous loci to measure genome instability events such as gross chromosomal rearrangements (GCR) and chromosome loss. Artificially constructed DNA repeats have also been validated as models to study genomic instability involving homologous recombination [23], [24]. To get further insight into the factors implicated in the maintenance of genome integrity we performed a screening of *S*. *cerevisiae* deletion strains for hyperrecombinant mutations, using different systems based on differentially transcribed DNA-repeats. We identified mutations that increase recombination in seven genes, four related with RNA metabolism, ranging from transcription to translation. Notably, among these mutations we found that deletion of *TRF4*, a polyA-polymerase of the TRAMP complex (Trf4/5-Air1/2-Mtr4 polyadenylation) that plays a role in RNA surveillance [25], [26], [27], confers a transcription-associated hyperrecombination phenotype that is mediated by the nascent mRNA. We provide genetic evidence that R-loops are formed in *trf4*Δ cells, such structures being responsible of the increase in recombination and mutation frequencies. Our data indicate that Trf4 is necessary for the maintenance of genome integrity, providing a link between mRNA surveillance and DNA metabolism in *S. cerevisiae.*

## Materials and Methods

### Strains and Plasmids

Yeast strains used are listed in Table 1. Plasmids pRS314L, pRS316L, pRS314LY, pRS316LY, pRS314SU, pRS316SU and pRS316-LYΔNS [28], pRS314L-*lacZ*, pRS314GL-*lacZ* [29], pGL-*rib^m^,* pGL-*Rib*+, pGAL:RNH1 [11] p413GAL1, p416-GAL1 [30] and p413GALAID [13] were used to determine recombination frequencies. Plasmid pCM184-LAUR was used for the analysis of mRNA expression levels as previously described [31]. Plasmids pNOPPATA1L, pNOPPATA1L-*TRF4*-WT and pNOPPATA1L-*TRF4*-*DADA* kindly provided by W. Keller, have been previously described [32].

**Table 1:** Table of Strains used in this work.

Strain	Genotype	Source/Reference
W303-1A	*MAT* **a** *ade2-1 can1-100 his3-11 leu2-3,112 trp1-1 ura3-1*	R. Rothstein
FY1679	*MATα ura3-52 his3*Δ*200 leu2*Δ*1 trp1*Δ*63*	Eurofan
FLRA006-01B(A)	*MAT* **a** *ura3-52 his3*Δ*200 leu2*Δ*1 LYS2 trp1*Δ*63 lsg1*Δ*::KAN*	Eurofan
FLPZ022-08B(AL)	*MAT* **a** *ura3-52 his3*Δ*200 leu2*Δ*1 LYS2 TRP1 rpl13A:: KAN*	Eurofan
FBS1008-02A(A)	*MAT* **a** *ura3-52 his3*Δ*200; leu2*Δ*1 LYS2 TRP1 tos3*Δ*:: KAN*	Eurofan
FPPROO3-03D(AL)	*MAT* **a** *ura3-52 his3*Δ*200 leu2*Δ*1 LYS2 trp1*Δ*63 apc9*Δ*:: KAN*	Eurofan
FSRM023-03C(A)	*MAT* **a** *ura3-52 his3*Δ*200 leu2*Δ*1 LYS2 TRP1 art1*Δ*:: KAN*	Eurofan
WFBE030	*MAT* **a** *ade2-1 can1-100 his3-11 leu2-3,112 trp1-1 ura3-1 trf4*Δ*:: KAN*	This study
WNOS032	*MAT* **a** *ade2-1 can1-100 his3-11 leu2-3,112 trp1-1 ura3-1 med2*Δ*:: KAN*	This study
TRF4D-C5	*MAT* **a** *ade2-1 can1-100 his3-11 leu2-3,112 trp1-1 ura3-1 trf4*Δ*:: KAN*	This study
MGY6-1A	*MAT* **a** *ade2 his3 trp1 ura3 leu2-k::ADE2-URA3::leu2-k*	[64]
AFGL-7D	*MAT* **a** *ade2 his3 trp1 ura3 leu2-k::ADE2-URA3::leu2-k lsg1*Δ*:: KAN*	This study
WFDL-1D	*MAT* **a** *ade2 his3 trp1 ura3 leu2-k::ADE2-URA3::leu2-k rpl13A*Δ*:: KAN*	This study
AFGL-2D	*MAT* **a** *ade2 his3 trp1 ura3 leu2-k::ADE2-URA3::leu2-k tos3*Δ*:: KAN*	This study
WFLR-2B	*MAT* **a** *ade2 his3 trp1 ura3 leu2-k::ADE2-URA3::leu2-k apc9*Δ*:: KAN*	This study
AFOR-1A	*MAT* **a** *ade2 his3 trp1 ura3 leu2-k::ADE2-URA3::leu2-k art1*Δ*:: KAN*	This study
AWT4-1C	*MAT* **a** *ade2 his3 trp1 ura3 leu2-k::ADE2-URA3::leu2-k trf4*Δ*:: KAN*	This study
MGY1-2D	*MAT* **a** *ade2 his3 trp1 ura3 leu2-k::ADE2-URA3::leu2-k med2*Δ*:: KAN*	This study

### Recombination and Mutation Analysis

Recombination frequencies were determined as described [33]. For each strain, the recombination frequencies are given as the average and standard deviation of the median recombination value obtained from fluctuation tests performed in 3–4 different transformants using 6 independent colonies per transformant. Recombinants were selected as Leu+ colonies for the plasmid containing *LEU2* truncated repeat systems. Recombination analyses for the chromosomal *leu2-k*::*ADE2-URA3*::*leu2-k* system (Lk-AU) were performed in wild-type and congenic mutants using 6 to 12 independent colonies grown in synthetic complete medium SC, and recombinants were selected in SC+FOA.

Mutation frequencies were determined in wild-type and mutant strains using the *Ptet*::*lacZ-URA3* (pCM184-LAUR) fusion construct. Ura^_^ mutants were selected in SC+FOA. The human *AID* gene, present in p413GALAID, was used for overexpression in 2% galactose medium. Median mutation frequencies were obtained by fluctuation tests performed in 3–4 different transformants using 6 independent colonies per transformant.

### Miscellaneous

β-galactosidase assays and Northern analyses were performed according to previously published procedures [31].

## Results

### New proteins involved in genome instability

**Figure 1:**
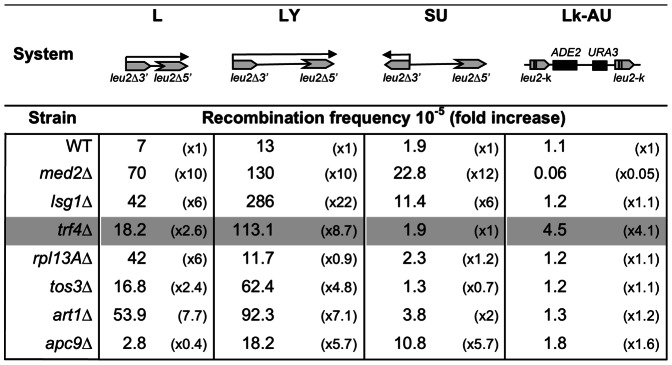
Recombination analyses of ***med2*** **,** ***lsg1, trf4, rpl13A, tos3, art1 and apc9*** **mutants.** A diagram of each recombination system (not drawn to scale) is shown at the top. Repeats are shown as gray boxes. Arrows indicate relevant transcripts produced by the constructs. For the L, LY and SU systems, recombination frequencies were determined in wild-type (FY1679) and mutant strains transformed with plasmids pRS314-L and pRS314-LY carrying the *leu2* direct-repeat systems, and pRS314-SU carrying an inverted repeats system. Recombinants were selected as Leu+. The average median value and SD of 3–4 fluctuation tests are shown. Recombination frequencies of *med2*Δ (MGY1-2D*)*, *lsg1*Δ (AFGL-7D), *trf4*Δ (AWT4-1C), *rpl13A*Δ (WFDL-1D), *tos3*Δ (AFGL-2D), *art1*Δ (AFOR-1A), *apc9*Δ (WFLR-2B) and wild-type *(*MGY6-1A*)* congenic strains carrying the chromosomal *leu2-k*::*ADE2-URA3*::*leu2-k* system are shown. For recombination analyses, independent colonies were obtained from SC and recombinants were selected in SC+FOA.

To identify novel genes with a role in genome stability, we performed a screening of S. *cerevisiae* deletion strains for hyperrecombinant mutants. We analyzed a total of 610 viable deletion strains constructed by the EUROFAN consortium. All strains were transformed with pRS314 and pRS216 centromeric plasmids carrying three different recombination systems, L, LY and SU, as described previously [28]. These systems are based on direct (L and LY) or inverted (SU) repeats of a 0.6 kb internal fragment of the *LEU2* ORF generated with two truncated copies of the *LEU2* gene (*leu2*Δ3′ and *leu2*Δ5′) spaced by different DNA sequences. Deletions (L and LY systems) and inversions (SU) were scored as Leu+ events and quantified by fluctuation tests. Among the strains analyzed, we found seven deletion mutants that conferred a hyperrecombinant phenotype (Figure 1). Four out of these mutants correspond to genes involved in RNA related processes: *MED2*, a subunit of the RNA polymerase II mediator complex [34]; *RPL13A,* a component of the large (60S) ribosomal subunit [35]; *LSG1,* a GTPase involved in 60S ribosomal subunit biogenesis [36], and *TRF4*, a component of the TRAMP complex involved in RNA surveillance [25], [26], [27]. The other three mutants were in *TOS3,* a redundant kinase that activates the Snf1/AMPK pathway that controls nutrient and environmental stress response [37]; *ART1,* involved in regulating the endocytosis of plasma membrane proteins [38], and *APC9,* involved in the regulation of protein stability [39]. Next, we measured the frequency of direct-repeat recombination in the chromosomal *leu2-k*::*ADE2-URA3*::*leu2-k* system. We constructed the different mutant strains carrying this chromosomal system and recombination leading to ura- deletions was scored. As shown in Figure 1, all mutants showed similar recombination frequencies to those of the wild-type strain, except *trf4*Δ. Thus we decided to focus our work on *trf4*Δ because it showed a hyperrecombination phenotype in all direct-repeat systems assayed, regardless of whether they were in plasmids or chromosomes.

### *trf4*Δ mutants confer transcription-dependent hyperrecombination

**Figure 2:**
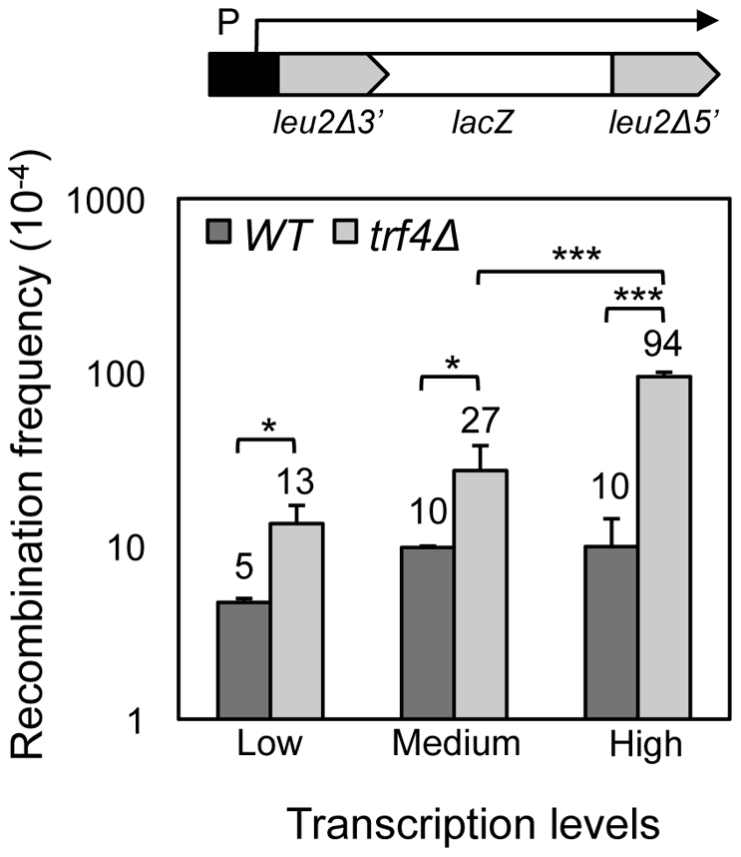
Effect of the level of transcription on the ***trf4Δ*** **hyperrecombination phenotype.** Isogenic strains W303-1A (WT) and TRF4D-C5 (*trf4Δ*) were transformed with plasmids pSCH204 (L-*lacZ* recombination system) or pRS314GL-lacZ (GL-*lacZ)* in which transcription is under the control of *LEU2* and *GAL1-10* promoters, respectively. Gray boxes represent *LEU2* repeats that flank the *lacZ* sequence. Arrow indicates the transcript produced. P. Promoter. Recombination frequencies are plotted as a function of the transcription levels. Low transcription refers to the GL-*lacZ* systems in strains cultured in 2% glucose; medium refers to L-*lacZ* in 2% glucose, and high to GL-*lacZ* in 2% galactose. The average median value and SD of 3-4 fluctuation tests are shown. Asterisks indicate statistically significant differences between the strains indicated, according to Student's t-tests (*, Ρ<0.05; ***, Ρ<0.0005).

We observed that the hyperrecombination phenotype of *trf4*Δ for the direct-repeat systems analyzed seems to be transcription-dependent (Figure 1). Recombination frequencies in *trf4*Δ strains were 2.6 and 8.7 times the WT levels for the L and LY systems, respectively. Both systems are based on the same direct repeats (an internal fragment of the *LEU2* gene) and differ in the length of the intervening sequence (31bp for L, and 5.57kb for LY) [28]. As in *trf4*Δ cells the recombination frequency is higher when there is a long DNA fragment transcribed between the two direct repeats, we wondered if deletion of *TRF4* indeed conferred a transcription-dependent genetic instability phenotype. To test this, we determined the effect of *trf4*Δ on recombination in the L-*lacZ* and GL-*lacZ* systems carrying 0.6-kb *leu2* direct repeats flanking the *lacZ* ORF under conditions of low (*GAL1* promoter in 2% glucose), medium (*LEU2* promoter) and high levels of transcription (*GAL1* promoter in 2% galactose). As can be seen in Figure 2, the higher the strength of transcription the stronger the increase in recombination. Altogether, the data indicate a statistically significant increase in recombination levels in *trf4*Δ cells respect to the wild-type that is transcription-dependent.

### The hyperrecombination phenotype of *trf4*Δ mutant is mediated by the nascent mRNA

**Figure 3:**
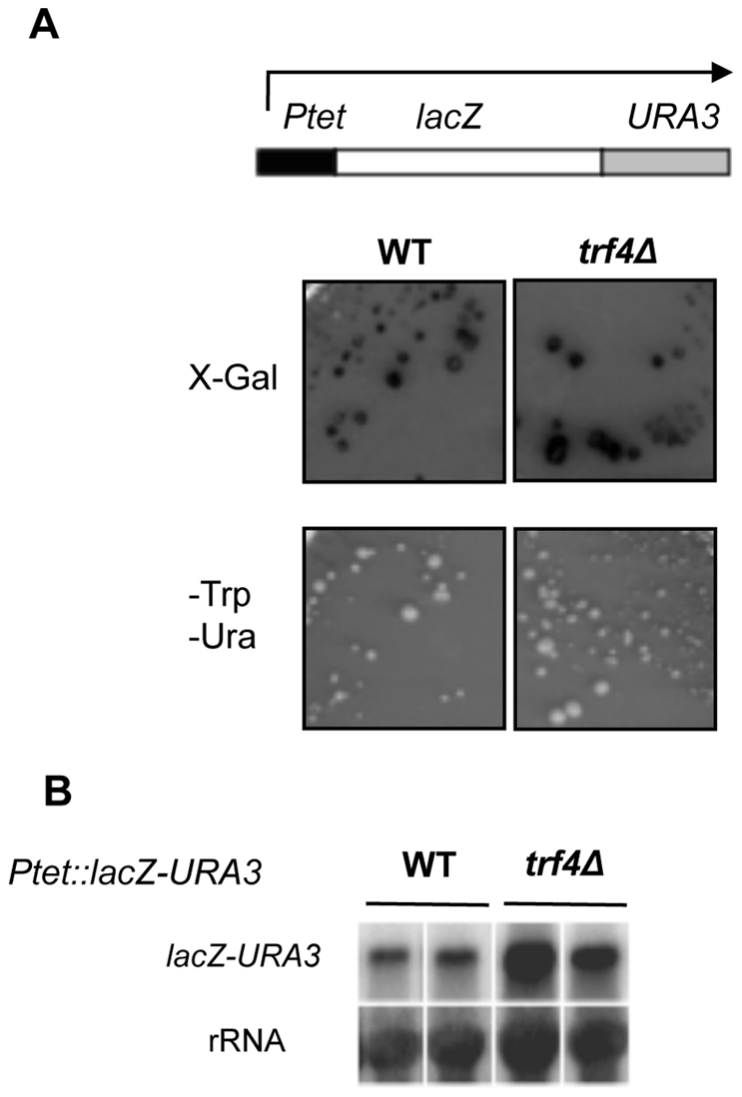
**Transcription analysis of the** ***trf4Δ*** **strain.** (**A**) Analysis of the ability of W303-1A and TRF4D-C5 (*trf4Δ*) strains carrying the *Ptet::lacZ-URA3* (LAUR) fusion construct (plasmid pCM184-LAUR) to form colonies on SC-trp-ura medium and to form blue colonies on SC-Trp complemented with X-Gal. (**B**) Northern analysis of the expression of the *Ptet::lacZ-URA3*. RNA was isolated from two different mid-log phase cultures from each strain, grown in SC-trp. We used the 3-kb *Bam*HI *lacZ* fragment and an internal 589-bp 25S rDNA fragment obtained by PCR, as probes.

The length and high GC content of *lacZ* gene makes transcription through this sequence poorly efficient in mutants impaired in transcription elongation [40], [41]. As *lacZ* transcription impairment was linked in many cases to hyperrecombination phenotype in mutants of THO and other mRNP factors [42], [43], we explored whether *lacZ* transcription was also affected in *trf4*Δ mutants. For this purpose, we analyzed gene expression in the LAUR expression system [31] that contains a 4.15-kb *lacZ-URA3* translational fusion under the control of the *Tet* promoter. Defects in *lacZ* expression were determined as poor growth in the absence of uracil and as lack of ß-galactosidase activity. As shown in Figure 3A *trf4*Δ cells behave as wild-type cells, suggesting that this mutant does not have a negative effect on transcription of the *lacZ-URA3* fusion. Moreover, northern analyses show that *lacZ* mRNA levels are much higher in *trf4*Δ mutants than in wild-type cells (Figure 3B), consistent with the previously described role of this protein in mRNA degradation [27].

**Figure 4:**
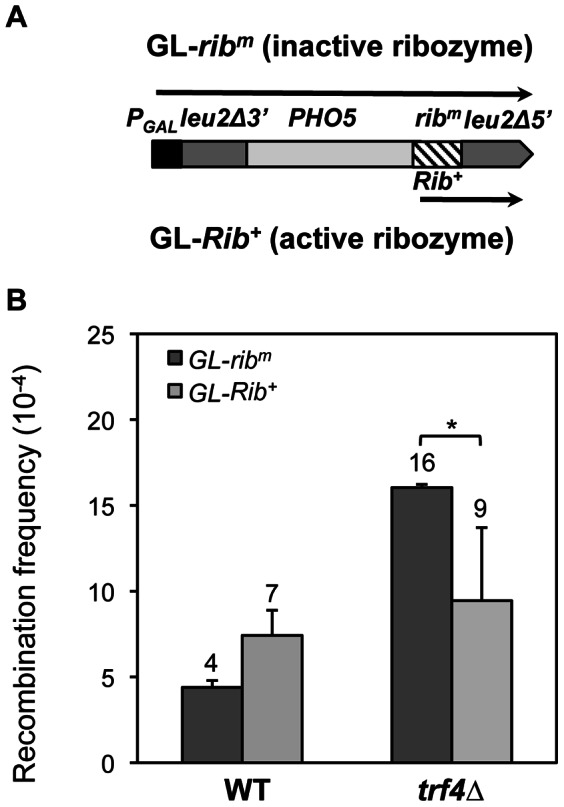
Nascent mRNA-dependency of the hyperrecombination phenotype of ***trf4*** Δ **mutants.** (**A**) Direct-repeat recombination systems GL-*Rib^+^* and GL-*rib^m^* containing the *PHO5-Rib^+^* or *PHO5- rib^m^* sequences flanked by two truncated copies of *LEU2* in direct orientation under the *GAL1* promoter. These systems contain respectively an active or inactive 52-bp ribozyme (*Rib*). The *rib^m^* system (inactive ribozyme) yields a long transcript, whereas in the *Rib^+^* system (active ribozyme) self-cleavage of the *PHO5-Rib* transcript leads to a shorter mRNA (represented by arrows). (**B**) Recombination frequencies in W303-1A (WT) and TRF4D-C5 (*trf4Δ*) cells containing the recombination systems GL-*Rib^+^* and GL-*rib^m^*. Experiments were performed in 2% galactose to allow expression of the direct repeats. The average median value and SD of 3–4 fluctuation tests are shown. Asterisk indicates statistically significant differences, according to Student's t-tests (*, Ρ<0.05).

Our data indicate that although *trf4* mutants show a transcription-dependent hyperrecombination phenotype (Figure 2), transcription seems not to be affected. Instead, higher amounts of mRNAs are accumulated, probably as a result of the defect in mRNA decay mediated by TRAMP. As the hyperrecombination phenotypes of several mRNP mutants depend on the nascent mRNA [11], [44], we analyzed whether this is the case for *trf4* mutants. We measured recombination in the GL-*Rib^+^* and GL*-rib^m^* repeats systems [11], which contain the sequence of the *PHO5* gene followed by either an active (Rib^+^) or inactive (rib^m^) hammerhead ribozyme respectively, located between two 0.6-kb-long *leu2* direct repeats (Figure 4A) under the control of the inducible *GAL1* promoter. Both the Rib^+^ and rib^m^ constructs synthesize a long mRNA, but upon transcription the active hammerhead ribozyme cleaves the transcript shortening the mRNA fragment still attached to the polymerase [11]. Figure 4B shows that in *trf4*Δ strains recombination levels in the GL-*Rib^+^* construct was lower than in GL*-rib^m^*, close to those of the wild type. The suppression of the hyperecombination phenotype by the ribozyme suggests that long nascent mRNAs contribute to the genetic instability in the absence of Trf4, therefore implicating that hyperrecombination was mediated by the RNA molecule.

### Hyperrecombination in *trf4*Δ cells does not depend on the catalytic polyadenylation domain of Trf4

**Figure 5:**
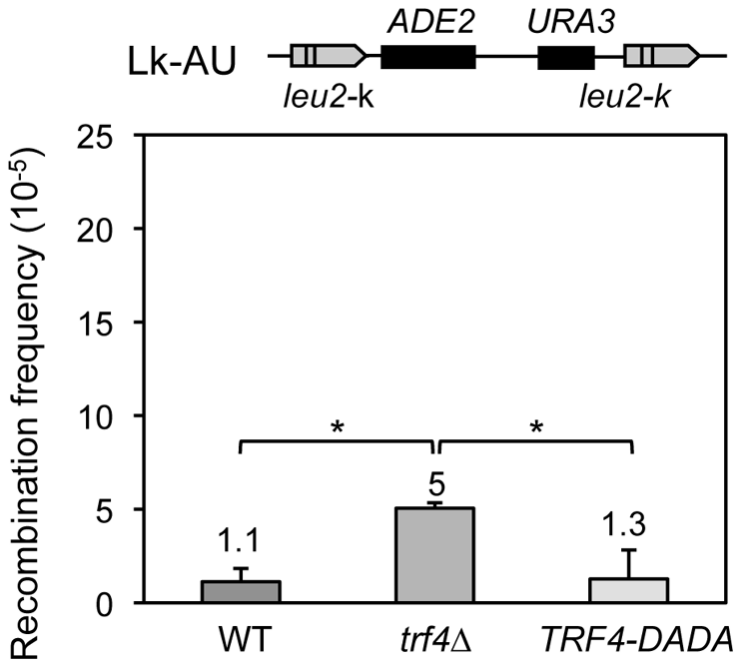
The hyperrecombination phenotype of ***trf4*** Δ **mutants is not dependent of its poly-adenylation catalytic domain.** Recombination frequencies in AWT4-1C cells carrying the chromosomal *leu2-k*::*ADE2-URA3*::*leu2-k* system and transformed with the pNOPPATA1L vector either empty (*trf4*Δ) or carrying the wild-type *TRF4* (WT) or the mutant *TRF4-DADA* alleles *(TRF4-DADA).* The average median value and SD of 3–4 fluctuation tests are shown. Asterisks indicate statistically significant differences, according to Student's t-tests (*, P<0.05).

As Trf4 is a cofactor of the TRAMP complex involved in RNA surveillance, we determined whether hyperrecombination was dependent on its polyadenylation activity. We measured the recombination frequency in *trf4*Δ cells carrying the chromosomal *leu2-k*::*ADE2-URA3*::*leu2-k* system transformed with a plasmid expressing either the wild-type *TRF4* allele or the polyadenylation-defective allele *TRF4*-*DADA* under the control of the *NOP1* promoter [32]. Trf4-DADA contains two aspartate to alanine mutations in the catalytic site of the polyA-polymerase that render the enzyme inactive [26]. Recombination levels were significantly reduced in *trf4*Δ cells by the overexpression of the *TRF4*-*DADA* allele, reaching values close to those of cells complemented with the wild-type *TRF4* allele (Figure 5). This suggests that hyperrecombination in *trf4*Δ cells takes place through a mechanism that is independent of its polyadenylation activity.

### Genetic instability in *trf4*Δ is mediated by R-loops

**Figure 6:**
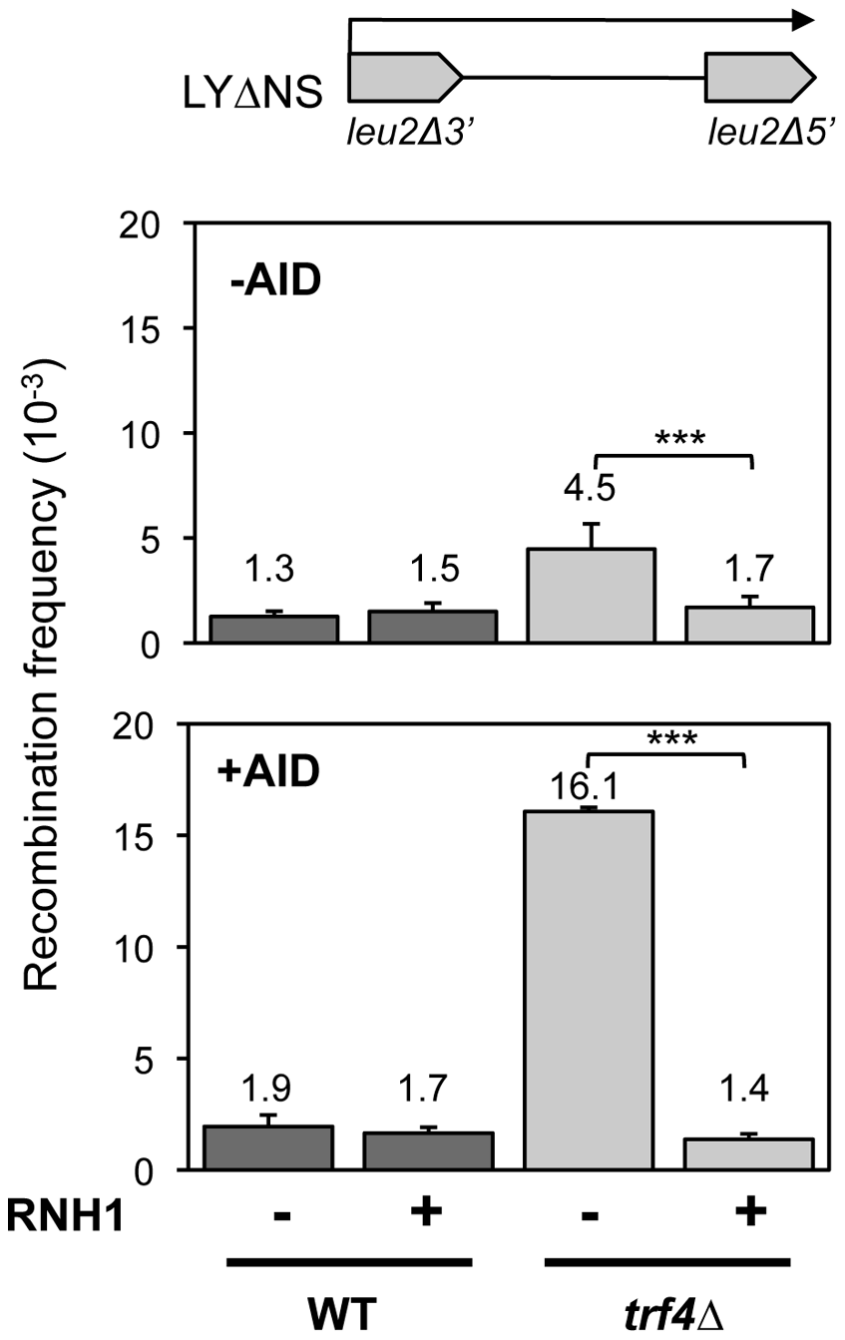
Genetic evidence for R-loop formation in ***trf4*** Δ **mutants.** Effect of RNaseH1 and AID over-expression on the mutation frequency in *trf4* mutants. Upper panel shows the analysis of recombination frequencies in W303-1A (WT) and TRF4D-C5 (*trf4Δ*) cells containing the recombination system LYΔNS, without RNaseH1 overexpression (-RNH1) or with over-expression of RNaseH1 (+RNH1). The latter was achivied with the multicopy plasmid pGAL-RNH1 carrying RNH1 under the GAL1 promoter. Lower panel shows recombination frequencies as in the upper one, but over-expressing AID from plasmid p413GALAID. The average median value and SD of 3–4 fluctuation tests are shown. Other details as in Figure 2. Asterisks indicate statistically significant differences, according to Student's t tests (***, Ρ<0.0005).

Next we determined whether the mRNA dependency of the hyperrecombination phenotype of *trf4*Δ cells was linked to the co-transcriptional formation of R-loops. To address this possibility we assayed the effect of RNase H overexpression in the *trf4*Δ mutant carrying the direct-repeat recombination system LYΔNS [28]. As can be seen in Figure 6 the hyperrecombination phenotype of *trf4*Δ was suppressed by the overexpression of RNase H.

**Figure 7:**
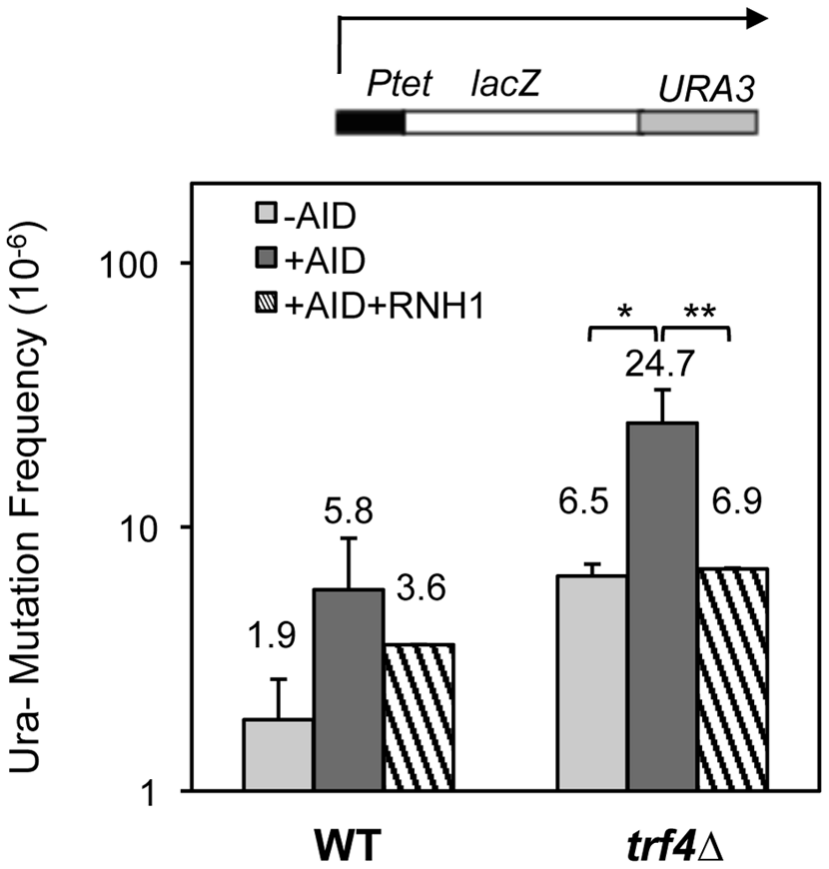
**Spontaneous and AID-induced mutation frequencies in wild-type and** ***trf4***Δ **strains.** Mutation frequency of W303-1A (WT), TRF4D-C5 (*trf4*Δ) strains, using the LAUR fusion construct. Ura^-^ mutants are selected in SC+FOA. The human *AID* gene was overexpressed in 2% galactose medium using plasmid p413GALAID. The median values of mutation frequencies and SD of 3–4 different fluctuation tests are shown. Asterisks indicate statistically significant differences, according to Student's t tests (*, P<0.05; **, Ρ<0.005).

As we have previously shown that as a consequence of R-loop formation expression of AID is able to strongly induce both mutation and recombination in yeast THO mutants [13], next we analyzed whether this was also the case for *trf4*Δ cells. As can be seen in Figure 6 AID expression increased recombination in *trf4*Δ mutants 8.2 times above the WT levels, which was suppressed by RNase H overexpression, consistent with the conclusion that R-loops are formed in *trf4*Δ mutants. To confirm this, we assayed the effect of AID expression on the mutation frequency in *trf4*Δ. We analyzed the frequency of Ura- mutations in the LAUR expression system. We observed that AID expression increased the frequency of Ura- colonies 3-fold in wild-type cells, consistent with previously reported data [13]. However this increase was of 13-fold in *trf4*Δ cells (Figure 7). As expected if this specific enhancement of *trf4*Δ was linked to R-loop formation, overexpression of RNase H reduced the frequency of Ura- mutations to values close to those of the wild-type (Figure 7). Altogether the data indicate that R-loops accumulate in the absence of *TRF4* and mediate the genomic instability of *trf4*Δ cells.

## Discussion

Here, we show the results of a screening for mutations that increased homologous recombination between repeated DNA fragments in yeast using genetic assays based on artificially constructed DNA repeats. We identified different mRNP biogenesis and transcription related proteins, as well as other factors, whose deletions lead to an increase in recombination in plasmid borne assays. We focused our studies in *TRF4,* a factor involved in RNA surveillance, because its absence causes a transcription-associated hyperrecombination phenotype both in chromosome and plasmid-borne systems. Using different genetic tools we show that this phenotype is dependent on the presence of the nascent mRNA and is mediated by R-loops. Our results therefore provide a new link between RNA quality control and genetic instability involving R-loops.

Our screening has permitted to identify a number of transcription and RNA related factors as suppressors of genome instability (Figure 1). These include *MED2*, one subunit of the transcription Mediator complex; *RPL13A*, a ribosomal subunit; *LSG1*, a GTPase involved in ribosomal biogenesis; and *TRF4*, a poly(A) polymerase of the TRAMP complex. Our results provide new evidence for the link between mRNA biogenesis and genome instability. Mutants affecting various steps of transcription, from initiation to termination, and RNA processing have been shown to lead to an increase of γH2A foci, YAC instability or hyperrecombination in yeast and human cells [14], [17], [19], [20], [31], [43], [44]. In addition, our screening identified other non-RNA related factors whose deletion could have an indirect impact on genome instability: *APC9*, encoding a subunit of the Anaphase-Promoting Complex/Cyclosome (APC/C), and *TOS3*, considered as the functional orthologous of LKB1, a mammalian kinase associated with Peutz-Jeghers cancer-susceptibility syndrome (Figure 1). Several studies have explored the role of LKB1 as a major actor of the AMPK/mTOR pathway connecting cellular metabolism, cell growth and tumorigenesis [45].

We have focused our interest in deciphering the genetic basis of genome instability in *trf4*Δ mutants. *TRF4* is a non-canonical polyA-polimerase that acts as a cofactor of the exosome complex for the quality control of different types of RNAs [46]. Interestingly, it was originally isolated in a synthetic growth screen with *top1* (topoisomerase one-requiring function) [47]. This is a notable observation because THO mutations also show a synthetic growth defect with *top1* [48] and indeed *hpr1*Δ was also recovered in that screen. In addition, *trf4* interacts genetically with mutations in different components of transcription, histone modification and histone remodeling complexes, and proteins involved in cohesion and DNA repair (reviewed in [49]). Beside its function in RNA surveillance, Trf4 has been shown to control chromatid cohesion, mitotic chromosome condensation and mitotic segregation [50], [51], [52], rDNA copy number [53], and telomere length [32]. Therefore, it seems that Trf4 plays a role in DNA metabolism of unknown nature.

We show that *TRF4* prevents genetic instability, as *trf4*Δ was identified as in our screening with recombination systems containing truncated repeats of the *LEU2* gene (Figure 1). Modulating the transcription levels of the repeats through constitutive and regulatable promoters, we have demonstrated that the hyperrecombination phenotype of *trf4*Δ is transcription-dependent (Figure 2). In addition, the hyperrecombination phenotype of *trf4*Δ cells can be suppressed by the action of a ribozyme inserted at the nascent mRNA as well as by RNase H overexpression (Figure 4 and Figure 6). The data indicate that RNA:DNA hybrids accumulate in the absence of this mRNA surveillance factor. Interestingly, we have previously reported that mutation in *RRP6*, the exonuclease subunit of the nuclear exosome, has an effect on transcription elongation and genome integrity [43]. Recently, it has been shown that deletion of *TRF4*, and of other mRNA surveillance factors, such as *KEM1* (an exonuclease involved in cytoplasmic mRNA decay), *AIR1* (a RNA-binding protein of the TRAMP complex) and *RRP6* lead to elevated GCRs in the form of terminal deletions and mini-chromosome losses using YACs. These events were partially suppressed by RNAse H overexpresssion [17], although its dependency on transcription was not established. Our results indicate that Trf4 is a factor that prevents different forms of genome instability, including that associated with transcription (Figure 1 and Figure 2). Importantly, we provide evidence that both recombination and mutation were enhanced in *trf4*Δ mutants (Figure 7). Altogether, our data suggest that cotranscriptional R-loop are responsible for both phenotypes, consistent with the exacerbated recombination and mutation phenotypes of *trf4*Δ cells upon AID cytidine deaminase overexpression (Figure 6 and Figure 7).

The impact of mutations in the RNA surveillance machinery on genome integrity reveals the global relevance of RNA metabolism in genome dynamics. Interestingly, in mammalian cells the core nuclear exosome subunit Rrp40 has been shown to be recruited to S regions of Ig genes and to be required for optimal class switching recombination [54]. It has been proposed that the exosome could provide the ribonuclease activity for degradation of the RNA strand of RNA-DNA hybrids exposing the template DNA strand to AID activity [54], but this is in principle unrelated with the AID-independent phenomenon described here. As AID induces mutation and recombination in different yeasts cells [55], [13], including *trf4* cells (this study) we believe that the loss of Trf4 may cause an accumulation of transcripts at the site of transcription with the potential to form R-loops, which are highly susceptible of AID, as previously shown for THO mutants [13].

R-loops structures accumulate in different mutants of mRNP biogenesis. mRNA processing and transcription factors could prevent R-loop formation by facilitating assembly of the nascent mRNA into a ribonucleoprotein particle, therefore limiting its ability to rehybridize with the template DNA strand (reviewed in [8]). Accumulative data indicate that R-loops in THO mutants hinder transcription elongation and generate recombinogenic structures that could represent an obstacle for the replication machinery [11], [56], [57], [58]. However, these properties are not shared by every mutant impairing mRNA biogenesis [43]. Indeed *trf4*Δ mutants do not show a reduction in the expression level the GC-rich *lacZ* gene from *E. coli* (Figure 3), in contrast to mutants of THO/TREX and other transcription elongation factors [31], [44]. On the other hand, mRNA processing and assembly into an export-competent mRNP is a tightly regulated process and a defect in mRNA processing can affect downstream steps [10], [59], [60]. TRAMP together with the nuclear exosome have been proposed to mediate a quality-control checkpoint activated upon mRNA export blockage [61]. Therefore, it is possible that aberrant mRNA transcripts that escape degradation in *trf4*Δ cells hybridize with the DNA contributing to R-loop formation and genome instability.

TRAMP plays a role in polyadenylation and stimulates RNA degradation mediated by the nuclear exosome [25], [26], [27]. However, polyadenylation is not essential for active degradation *in vitro* [27] and a polyadenylation-defective Trf4 protein is fully active, suggesting that mRNA degradation triggered by Trf4 is independent of its polyadenylation activity [61]. Indeed, genome-wide expression analysis shows that the overexpression of *TRF4-DADA* restores the levels of most RNAs with an altered expression in *trf4*Δ cells, except a small fraction corresponding to highly expressed and structured RNAs [32]. The fact that hyper-recombination phenotype of *trf4*Δ cells is suppressed by the overexpression of the *TRF4*-*DADA* mutant allele (Figure 5) indicates that occurs via a polyadenylation-independent mechanism. Interestingly, other DNA-related phenotypes, such as the maintenance of telomere, have also been shown to be polyadenylation independent [32]. In addition, although Trf4 has been defined as a non-canonical poly-A-polymerase playing a role in RNA surveillance, its function role is not restricted to RNA degradation, but rather contributes to the processing of different RNAs such as tRNAs, snoRNA, snRNAs and rRNA precursors [32], [62], [63]. Indeed, Trf4 has been shown recently to be associated with introns *in vivo,* as shown by crosslinking-RNA-immunoprecipitation, and to regulate degradation of spliced-out introns [32]. Therefore Trf4 could link RNA processing with the maintenance of genome integrity.

A number of reports suggest that R-loops are formed at a higher frequency in the genome than previously anticipated with an impact in both gene expression and genome integrity [8]. Given the role of Trf4 in the processing and degradation of different types of RNAs [46], it is also possible that the loss of rDNA repeats observed previously in *trf4*Δ cells, could be associated with the formation of R-loops [53], [15]. In summary our work suggests that *trf4*Δ leads to a general transcription-associated genome instability phenotype that is mediated by the cotranscriptional formation of R-loops, providing a further connection between genome dynamics and RNA metabolism.
